# Ischemia modified albumin: does it change during pneumoperitoneum in robotic prostatectomies?

**DOI:** 10.1590/S1677-5538.IBJU.2014.0677

**Published:** 2016

**Authors:** Serpil Ustalar Ozgen, Bora Ozveren, Meltem Kilercik, Ugur Aksu, Binnaz Ay, Ilter Tufek, Ali Riza Kural, Levent N.Turkeri, Fevzi Toraman

**Affiliations:** 1 Department of Anesthesiology and Reanimation, Acibadem University, Istanbul, Turkey;; 2 Department of Urology, Acibadem University, Istanbul, Turkey;; 3 Acibadem Labmed, Istanbul, Turkey;; 4 Department of Biology, Faculty of Science, Istanbul University, Istanbul, Turkey;; 5 Department of Anesthesiology, Acibadem Maslak Hospital, Istanbul, Turkey;; 6 Clinics of Urology, Acibadem Maslak Hospital, Istanbul, Turkey;; 7 Clinics of Urology Acibadem Kadikoy Hospital, Istanbul, Turkey

**Keywords:** Ischemia, Albumins, Head-Down Tilt, Robotics, Prostatectomy

## Abstract

**Background:**

The unique positioning of the patient at steep Trendelenburg with prolonged and increased intra-abdominal pressure (IAP) during robotic radical prostatectomy may increase the risk of splanchnic ischemia. We aimed to investigate the acute effects of IAP and steep Trendelenburg position on the level of ischemia modified albumin (IMA) and to test if serum IMA levels might be used as a surrogate marker for possible covert ischemia during robotic radical prostatectomies.

**Patients and Methods:**

Fifty ASA I-II patients scheduled for elective robotic radical prostatectomy were included in this investigation.

**Exclusion criteria:**

The patients were excluded from the study when an arterial cannulation could not be accomplished, if the case had to be converted to open surgery or if the calculated intraoperative bleeding exceeded 300ml. All the patients were placed in steep (45 degrees) Trendelenburg position following trocar placement. Throughout the operation the IAP was maintained between 11-14mmHg. Mean arterial blood pressure (MAP), cardiac output (CO) were continuously monitored before the induction and throughout the surgery. Blood gases, electrolytes, urea, creatinine, alanine transferase (ALT), aspartate transferase (AST) were recorded. Additionally, IMA levels were measured before, during and after surgery.

**Results:**

(1) MAP, CO, lactate and hemoglobin (Hb) did not significantly change in any period of surgery (p>0.05); (2) sodium (p<0.01), potassium (p<0.05) and urea (p<0.05) levels decreased at postoperative period, and no significant changes at creatinine, AST, ALT levels were observed in these patients; (3) At the end of surgery (180 min) pCO_2_, pO_2_, HCO_3_ and BE did not change compared to after induction values (p>0.05) but mild acidosis was present in these patients (p<0.01 vs. after induction); (4) IMA levels were found to be comparable before induction (0.34±0.04), after induction (0.31±0.06) and at the end of surgery (0.29±0.05) as well.

**Conclusion:**

We did not demonstrate any significant mesenteric-splanchnic ischemia which could be detected by serum IMA levels during robotic radical prostatectomies performed under steep Trendelenburg position and when IAP is maintained in between 11-14 mmHg

## INTRODUCTION

Robot assistance in laparoscopic surgery has undoubtedly contributed to the advancement of minimally invasive oncologic surgery. Robotic surgery provides a number of potential benefits such as improvement in surgical precision, diminished blood loss, reduced postoperative pain, improved cosmetic outcome, shorter convalescence and hence improved patient satisfaction (1, 2). Robot assisted laparoscopic radical prostatectomy (RARP) is the most frequently performed robotic procedure in urology. Despite the established advantages, there are several important issues related to the intra-operative management specific to this procedure. The positioning of patients during RARP impacts the risks related to hemodynamic changes such as increased systemic vascular resistance (SVR), mean arterial pressure (MAP), filling pressures and reduction in cardiac index (CI) (3, 4). High intra-abdominal pressure (IAP), especially if over 15mmHg, increases cerebral blood flow and intracranial pressure, while decreasing portal, hepatic vein flow and the total hepatic microcirculation (5-8). Increased IAP furthermore decreases mesenteric blood flow and impedes gastrointestinal microcirculation (8, 9). The pneumoperitoneum (PP) similarly leads to decreased arterial and venous flow in renal medulla and cortex (10-12).

The fixed positioning of the patients followed by the docking of the robot in steep Trendelenburg position and the relatively long duration of this procedure can thus cause excessive mechanical pressure over the gastrointestinal, respiratory and the cardiovascular systems, increase the risk of hypothermia, intensify the hemodynamic and respiratory adverse effects of the PP and may as well give rise to mesenteric-splanchnic hypoxic ischemia (4).

Serum ischemia modified albumin (IMA) is a new, FDA approved biomarker of ischemia, and increases in patients with acute coronary syndrome (13-16). IMA is produced during an ischemic attack and is present in blood in easily detectable concentrations (13, 17-19). Hypoxia, acidosis and free radical production reduce the ability of human serum albumin to bind metals like cobalt to its N-terminus, which in turn causes IMA production. Endothelial or extracellular hypoxia, acidosis, and free oxygen radicals have been shown to cause IMA increase (20, 21), thus IMA can be detected early on the beginning of ischemia. Moreover IMA was found to increase in patients with mesenteric ischemia (22). It has been identified as a helpful marker for determining the alterations in the splanchnic and the visceral blood flow during laparoscopic cholecystectomies (23).

After myocardial ischemia, the serum levels of IMA rise within minutes and continue to increase for 6-12 hours, after which they return to normal (24-28). Increase in IMA concentrations has also been shown to indicate tissue ischemia in other conditions such as peripheral vascular disease, exercise-induced skeletal muscle ischemia, end-stage renal disease patients on haemodialysis, acute stroke, calf-muscle ischemia (29-33).

In the present study, we aimed to investigate the acute effects of IAP and steep Trendelenburg position on the level of ischemia modified albumin (IMA) and to test if serum IMA levels might be used as a surrogate marker for possible covert ischemia during robotic radical prostatectomies.

## MATERIALS AND METHODS

Local ethics committee approval for this study (ATADEK No: 2013-456/B.30.2.ACU.0.00.00.050-06) was provided by ATADEK, Acibadem University, Ethics Committee, Istanbul, Turkey on 01 February 2013.

Fifty male patients, aged 55-75 years, ASA I-II, scheduled for RARP were included in the study and informed consents were taken.

Exclusion Criteria: The patients were excluded from the study when an arterial cannulation could not be accomplished, if the case had to be converted to open surgery or if the calculated intraoperative bleeding exceeded 300ml.

### General Procedure

All patients were pre-medicated with midazolam 0.05mg/kg intravenously (i.v.) and standard monitorization, including, electrocardiography (ECG), invasive blood pressure (IBP), pulse oximetry (SpO_2_), regional cerebral oxygenation (rSO_2_), cardiac output (CO), and end tidal carbon dioxide (ETCO_2_), was applied. Anaesthesia induction was performed by propofol 2.5-3.5mg/kg, remifentanil 1mic/kg i.v. and muscle relaxation was performed by rocuronium bromide 0.6mg/kg i.v. bolus and infusion 4-15mcg/kg/min. Anaesthesia was maintained by remifentanil 0.025-0.05mic/kg/min i.v.infusion and by sevoflurane 0.8-1% in O_2_:N_2_O/40:60 in all patients. PEEP was adjusted between 4.6-4.8mmHg. The patients were placed in steep Trendelenburg position (45 degrees head-down angulation) after trocar placement and until the robot was un-docked. The IAP was maintained between 11-14mmHg throughout the laparoscopic stage of the operation.

MAP and CO were measured constantly as systemic hemodynamic parameters and recorded at particular instants: (1) before induction (BI); (2) after induction (AI); (3) 5^th^min; (4) 60^th^min; (5) 90^th^min; (6) 120^th^min; (7) 150^th^min; (8) 180^th^min of PP. Arterial blood levels of lactate, hemoglobin, pH, pCO_2_, pO_2_, HCO_3_^-^ and base access were monitored at same intervals. Additionally, serum sodium, potassium, blood urea nitrogen (BUN), creatinine, alanine transferase (ALT) and aspartate transferase (AST) were assessed preoperatively and postoperatively. Blood was sampled for interim measurements of serum ischemia modified albumin levels before induction (BI), after induction (AI) and the end of surgery (ES). The serum samples were stored at -20 degrees until they were sent to laboratory for IMA quantification. All of the serum samples for IMA measurements remained intact.

### Determination of Ischemia modified Albumin (IMA)

IMA levels were determined according to the method defined by Bar-Or et al. (24). Briefly, 200uL serum was added to 50uL 0. 1% (w/v) cobalt chloride (Sigma Aldrich, St. Louis, MO; CoCl_2_.6H_2_O). After gentle shaking, 10 minutes were waited to allow cobalt binding to albumin. Then 50uL dithiothreitol (DTT) (Sigma) was added as a colouring agent. As control, 50uL of distilled water was used instead of DTT. After 2 minutes, 1mL of 0.9% NaCl was added to stop the reaction, and the absorbance at 470nm was determined using a spectrophotometer (24). The difference of absorbance units between control and DTT samples were recorded. The results were quantified as absorbance unit (ABSU) and values greater than 0.400 ABSU were accepted as showing lower binding capacity for cobalt, therefore indicative of ischemia, whereas values lower than 0.400 ABSU were interpreted as lack of ischemia (24, 25).

### Statistical analysis

Outcomes were reported as the mean±SEM. Statistical analysis was performed using GraphPad Prism version 5.0 for Windows (GraphPad Software, La Jolla, Calif). Results were compared using repeated measures ANOVA-tukey post hoc test used and a p-value of <0.05 was considered statistically significant.

## RESULTS

The descriptive characteristics of patients and duration of surgery is summarized in Table-1.

**Table 1:** Characteristics of the patients and duration of pneumoperitoneum(PP).

Age	Height (cm)	Weight (kg)	BSA	PP. duration (min.)
59.7±1.3	171.2±3.0	81.9±1.7	1.95±0.04	186.5±6.54

(Acibadem Kadikoy /Maslak Hospital, 2013)

### Systemic hemodynamics results

**Figure 1:**
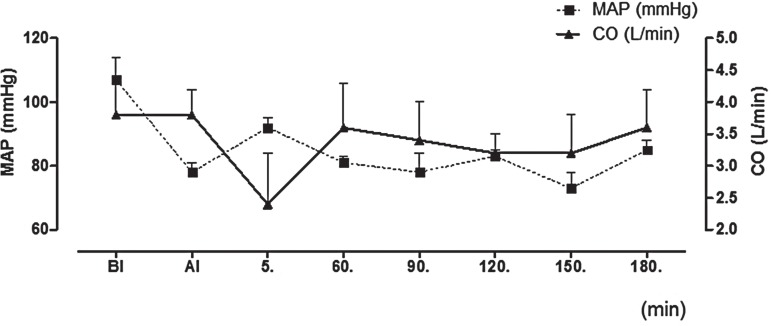
Systemic hemodynamic parameters.

The systemic hemodynamic values are presented in Figure-1. Induction of anaesthesia did not cause a statistically significant effect on MAP (p>0.05). Likewise, CO values were not affected by anaesthesia. At any time of surgery, both MAP and CO values were found to be similar compared to their respective levels after anaesthesia induction (p>0.05).

### Blood gases analysis results

**Figure 2:**
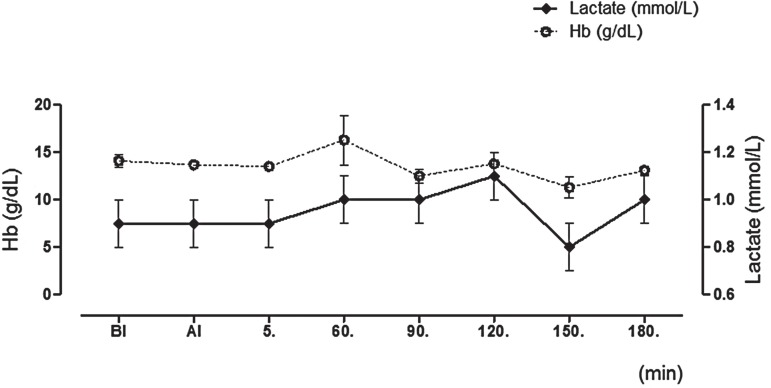
Lactate and hemoglobin values at all-time points.

Blood gasses and related parameters are presented in Figure-2 and Table-2. The arterial pH was more acidotic at the 150^th^ and 180^th^min of PP when compared to 5^th^min of PP. pCO_2_ values were also found higher concurrently. pO_2_ values were higher preoperatively than the pre-induction values during PP. While HCO_3_^-^ level was not statistically different, base excess (BE) levels were higher at 60^th^, 120^th^ and 180^th^min. Hemoglobin and lactate values were not statistically altered during the operation.

**Table 2:** Blood Gases parameters.

	BI			AI			5^th^ min			60^th^ min			90^th^min			120^th^min			150^th^ min			180^th^min		
pH	7.41	±	0.02	7.41	±	0.01	7.42	±	0.01	7.42	±	0.01	7.37	±	0.01	7.4	±	0.01	7.33c	±	0.03	7.36	±	0.01^aabbbcccdd^
**pCO**_**2**_	37.8	±	2.8	35.6	±	0.7	33	±	0.5	32.5b	±	0.6	39.2	±	2.3	34.3	±	0.6	43.3	±	2.3^bcc^	38.5	±	1.4^bbbcccd^
**pO**_**2**_	140.2	±	37.3	211.6	±	14	157	±	8.1^aaa^	153	±	5.9^aaa^	145	±	16.2	160.8	±	5.6^aa^	128	±	8.4	154.7b	±	7.4
**HCO**_**3**_^**-**^	25.2	±	0.7	22.6	±	0.3	21.6	±	0.3	24.6	±	3.8	22.8	±	0.7	21.2	±	0.3	22.3	±	0.9	21.5	±	0.4
**BE (-)**	0.8	±	1.0	1.3	±	0.3	1.6	±	0.3	2.4a	±	0.3	1.4	±	0.7	2.3a	±	0.3	2.3	±	1.2	2.4a	±	0.4

(**BI =** Before induction, **AI =** After induction, 5^th^ min: 5^th^ min of PP) (^aa^p<0.01, ^aaa^p<0.001 vs AI; ^b^p<0.05,^bbb^p<0.001 vs PP 5^th^ min;^cc^p<0.01, ^ccc^p<0.001 vs PP 60^th^ min;^d^p<0.05 vs PP 120^th^ min) (Acibadem Kadikoy /Maslak Hospital, 2013)

### Routine biochemistry and plasma electrolyte results

Plasma ions, urea, creatinine, ALT, AST levels are presented in Table-3. Postoperative levels of sodium, potassium and urea were lower (p<0.01, p<0.05, p<0.05; respectively), whereas, postoperative levels of creatinine, ALT and AST were not different from the preoperative values (p>0.05).

**Table 3:** Routine blood chemistry parameters .

	Pre-op			Post-op		
**Na+ (mmol/L)**	140	±	0	138.2	±	0.4**
**K+ (mmol/L)**	4.5	±	0.1	4.1	±	0.1*
**Urea (mg/dL)**	31.7	±	1.3	24.2	±	3.7*
**Creatinine (mg/dL)**	0.9	±	0	0.8	±	0.1
**AST (U/L)**	22.9	±	1.1	19	±	1.9
**ALT (U/L)**	24.7	±	1.9	21.7	±	4.1

(*p<0.05,**p<0.01vs Pre-op)

(Acibadem Kadikoy /Maslak Hospital, 2013)

### IMA levels results

**Figure 3:**
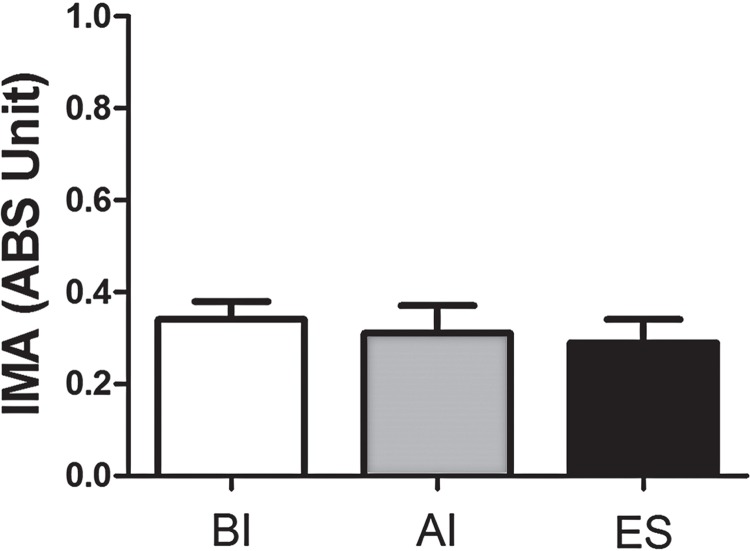
Serum IMA levels at three time points (BI: Before induction, AI: After induction, ES: End of surgery).

IMA assessments at three time intervals are presented in Figure-3. Mean IMA (ABS unit) values were 0.34±0.04, 0.31±0.06 and 0.29±0.05. The variances between intervals were not found to be statistically significant (p>0.05) (Figure-3).

## DISCUSSION

RARP is performed while the patient is uniquely placed in a 45 degrees head-down (Trendelenburg) position and requires CO_2_ insufflation to maintain an IAP of 12-15mmHg. We designed this study to investigate a hypothesis that even with a standard IAP, some form of mesenteric-splanchnic injury might be induced due to an extra gravitational pressure or a traction force caused by the exclusive fixed patient-positioning in addition to the duration of robot-assisted laparoscopic radical prostatectomy. We have come up with a hypothesis of a possible mesenteric-splanchnic injury during RARP subsequent to a simple observation of frequent and prolonged post-operative ileus in patients undergoing this operation. Our purpose was to assess the acute effects of IAP at steep Trendelenburg position during this operation by hemodynamic monitorization, blood gas and electrolyte analyses and utilizing serum IMA as a biomarker for a supposedly overlooked mesenteric-splanchnic ischemia during robotic prostatectomies. However, our research failed to suggest any significant association of this exclusive patient positioning or variances of IAP levels with mesenteric-splanchnic ischemia as assessed by means of serum IMA during robotic radical prostatectomy.

Previous studies evaluating the course of cardiovascular changes during PP pointed out that the CI gradually increased and systemic vascular resistance decreased 10 minutes after CO_2_ insufflation (35-37). Additionally other studies suggested that CO rate decreased by 10-30% in Trendelenburg position (37-39). On the other hand, the mean CO level stayed stable (between 2.4-3.8L/min) in our study. It decreased to 2.4L/min at 5 minutes of PP but this change was statistically insignificant.

Lestar et al. examined the circulatory effects of an extreme Trendelenburg position (45º) on patients during robot-assisted laparoscopic radical prostatectomy and reported an almost 3-fold increase in central venous pressure compared with the initial value. MAP was increased by 35% in this study whereas heart rate (HR), stroke volume (SV), CO, and mixed venous oxygen saturation were unaffected during surgery, as were echocardiographic heart dimensions. In the horizontal position after PP exsufflation, filling pressures and MAP returned to baseline levels (38).

In another study designed to evaluate hemodynamic changes associated with head-down positioning and prolonged PP during RARP, invasive hemodynamic parameters were measured by transpulmonary arterial thermodilution using the PiCCO system with a femoral artery catheter, CI, HR, MAP, systemic vascular resistance index, intrathoracic blood volume, and central venous pressure were recorded with the patient in the supine position, after head-down tilt, intraoperatively after 30 min, 1h, 2hs, 3hs, and 4hs of PP at an insufflation pressure of 12mmHg. Placing the patient in the Trendelenburg position caused a significant increase in CVP, whereas all other hemodynamic parameters remained nearly unaffected. The induction of PP resulted in a significant increase in MAP whereas no other parameter was affected. Even at 4 hours of PP, only mild hemodynamic changes were observed (38). In our study, MAP was lower compared to pre-induction values throughout the operation, except a slight but statistically insignificant increase after PP. In our study, we did not prefer the use of invasive cardiac control.

In the present study, the IAP was 14mmHg at the start of PP and then maintained around 11mmHg for a mean duration of 3 hours. Post-operative levels of AST, ALT were not statistically different compared to the preoperative values. In an animal study assessing the effect of prolonged PP on liver function and perfusion, it was found that the liver sustained no damage due to prolonged PP during laparoscopic surgery. In that study, the IAP was maintained at 14mmHg and the mean operation time was 6 hours (34).

In our study Group, the blood pH values were significantly lower at 150^th^min and 180^th^ min of PP than other intervals. At the same periods, pCO_2_ was also significantly higher compared to those measurements at other times. This can be explained by increased CO_2_ absorption during that time intervals of PP. In a study assessing the acid-base status and hemodynamic changes during PP, it was reported that there was a significant absorption of CO_2_ gas across the peritoneum which caused substantial acidemia and hypercapnia, which is also consistent with our results. In that study by Ho et al., the IAP did not affect metabolic function, acid-base balance or hemodynamics (33). The investigators installed an IAP of 7 and 14mmHg each for 30 minutes to test the IAP ranges for laparoscopic procedures which elicited splanchnic and pulmonary hemodynamic and metabolic changes (33). The effect of low IAP (7mmHg) on splanchnic perfusion was pointed out to be minimal whereas higher IAPs (14mmHg) decreased the portal and hepatic blood flow, lowered the hepatic and intestinal tissue pH. In our study, the IAPs were kept in the range of 11-14mmHg throughout the operations and we found no statistically significant increase in IMA levels, which would have indicated ischemia.

In a study evaluating the effects of PP on mesenteric ischemia-reperfusion injury by measuring intestinal tissue oxygen pressure (Pt_i_O_2_) and oxidative damage during laparoscopic and open colon surgery, the authors found that during laparoscopic surgery, there was a significant decrease of Pt_i_O_2_ only when PP was increased to 15mmHg. Although malondialdehyde (MDA) significantly increased in both Groups after mesentery traction and at the end of operation versus baseline levels, there was no difference between techniques (39). When the effects of prolonged PP (4 hours) during RARP were investigated, it was found that MDA concentrations were significantly elevated at various intervals as compared with the pre-insufflation value and also the intra-mucosal pH value decreased significantly after CO_2_ insufflation compared with the pre-insufflation values. It was concluded that prolonged PP in RALP resulted in decreased splanchnic blood flow and PP itself produced oxidative stress (40). In our study, PP lasted 3.5 hours and the difference between pre- and postoperative IMA values was not statistically significant. Therefore, no ischemia was detected by measuring IMA.

Our findings did not reveal any renal injury as there was no significant statistical difference between the preoperative and postoperative BUN and creatinine values, unlike the study of Bishara et al., where renal perfusion and function were decreased by induction of IAP of 14mmHg (41).

The maintenance of the IAP within 11-14mmHg during the RARP operations is necessary in order to avoid the complications of PP. This safety measure certainly protects the patients from the adverse effects of high IAP. The restriction of IAP might be considered a limitation for this clinical study, and the sensitivity of IMA as an ischemic biomarker can further be tested by designing animal studies where IAP may be increased to higher levels. Further studies may as well be performed utilizing novel biomarkers to identify whether there are any mesenteric compartment like syndromes during RARP.

Based upon the findings obtained as a result of current study; although patients were kept in steep (45 degrees) Trendelenburg position during robotic prostatectomies, IAP between 11-14mmHg does not cause any hypoxia/ischemia detected by conventional measurement techniques. Even if there is a hypoxia, which is out of detection limits, this cannot be followed by serum IMA levels.

In conclusion, during robotic radical prostatectomies performed under steep Trendelenburg position and when IAP is maintained in between 11-14mmHg, we did not demonstrate any significant mesenteric-splanchnic ischemia that could be detected by serum IMA levels.
